# Animal models of focal brain ischemia

**DOI:** 10.1186/2040-7378-1-7

**Published:** 2009-11-13

**Authors:** Kenneth M Sicard, Marc Fisher

**Affiliations:** 1 Department of Neurology, University of Massachusetts Medical School, 55 Lake Avenue North, Worcester, MA 01453, USA

## Abstract

Stroke is a leading cause of disability and death in many countries. Understanding the pathophysiology of ischemic injury and developing therapies is an important endeavor that requires much additional research. Animal stroke models provide an important mechanism for these activities. A large number of stroke models have been developed and are currently used in laboratories around the world. These models are overviewed as are approaches for measuring infarct size and functional outcome.

## Utility of Animal Models of Ischemic Stroke

Stroke is the third leading cause of death and a major cause of disability in the United States [1,2]. Each year, there are approximately 731,000 new strokes and half of the survivors suffer from permanent handicap [3]. Stroke costs the United States approximately $50 billion annually in direct and indirect costs [4]. Given these facts, stroke is a major public health issue requiring urgent development of effective therapies: experimental models of focal brain ischemia help in achieving this goal.

80% of human strokes are ischemic in origin [2]. Thus, experimental models of focal cerebral ischemia have been developed in an attempt to closely mimic the changes that occur during and after human ischemic stroke. These models are used to discover the mechanisms involved in the evolution of ischemic injury which, in turn, can lead to the development of novel therapeutic strategies for stroke. These same animal models can then be used to test the safety and efficacy of these treatments *in vivo*.

Most human ischemic strokes are caused by occlusion of the middle cerebral artery (MCA) [5] and so animal models were developed to induce ischemia in this arterial territory. These models aim to satisfy the following criteria: (1) to mimic the pathophysiological changes found in human stroke, (2) to create reproducible lesions, (3) to employ procedures that are relatively simple and noninvasive, (4) to be of low financial cost, and (5) to enable monitoring of physiologic parameters and analysis of brain tissue for outcome measures [6].

Many higher animal species fulfill the aforementioned stroke modeling criteria; however, rats are the most commonly used animals for several reasons, including: (1) their resemblance to humans in their cerebral anatomy and physiology, (2) their small size which enables easy analysis of physiology and brain tissue, (3) their low cost, (4) the remarkable genetic homogeneity within strains, and (5) greater public and institutional ethical acceptability of use relative to larger animals. Thus, the remainder of this chapter will focus primarily on rat models of ischemic stroke [7-9].

## Permanent versus Transient Ischemia

Focal brain ischemia models can be categorized into two groups: permanent and transient ischemia. Permanent ischemia results in a region of severe ischemic damage (core) surrounded by a zone of less damaged tissue [10]. Reestablishment of perfusion after 3 hours does not reduce infarct size in all animal models [11].

Transient focal ischemia produces varying degrees of ischemic damage depending on the duration of ischemia. In rats, as little as 8 minutes of ischemia causes selective neural necrosis and ischemia for more than 30 minutes is always associated with infarction [12]. Importantly, after transient ischemia, brain damage results from both the ischemia and the effects of reperfusion (reperfusion injury). Compared to permanent occlusion, which mimic only a minority of human strokes where there is no recanalization, transient models better correlate with conditions such as therapy-induced thrombolysis, spontaneous thrombolysis, and transient ischemic attack. However, both permanent and transient ischemia models are needed prior to clinical drug development studies because of the heterogeneity of human stroke [13,14].

Long-term studies are becoming increasingly important in translational stroke research; thus, the animal survival rate should be considered when designing an experimental protocol. Generally, regardless of the employed technique, models of transient ischemia offer higher survival rates relative to permanent occlusion and thus may be more suited for long-term studies. Additionally, survival is enhanced by proper surgical technique, maintaining physiological parameters within normal ranges during experimentation, and attention to animal nutrition, among other things. For studies of neuroprotection and/or thrombolysis, the effects of putative therapies on survivability must also be taken into consideration.

## Rat Models of Focal Cerebral Ischemia

Many animal models of focal cerebral ischemia exist. In this chapter, special focus is paid to the intraluminal MCA occlusion and embolus models because of their relatively widespread use in the development of treatments for stroke. Importantly, these methods can also be performed on mice, allowing for transgenic studies of the pathophysiology of stroke.

### Intraluminal MCA Occlusion Model

This model was originally described by Koizumi *et al *and has since been modified by others [11]. It is the most commonly used of rat models of stroke due to its relative simplicity and noninvasiveness. The MCA is occluded by inserting a monofilament suture into the internal carotid artery (ICA) to block blood flow to the MCA either permanently or transiently by keeping the filament in place or withdrawing it, respectively. Several manuscripts describe in detail the technical and procedural features of this model [11-16].

This model typically induces infarcts in the lateral caudatoputamen and frontoparietal cortex [17]. The infarct is reproducible and there is a significant ischemic penumbra early after MCA occlusion, making this model suitable for testing neuroprotective agents [18]. However, several technical factors may influence infarct size, such as: 1) physical differences in the employed monofilament suture, (2) insertion distance of the suture, and (3) accidental premature reperfusion [19-21]. It is therefore essential that standardized surgical and technical procedures be used by adequately trained personnel in order to generate reproducible lesions. There are also some complications with the intraluminal MCA occlusion model such as subarachnoid hemorrhage secondary to suture-induced arterial rupture, spontaneous hyperthermia when the duration of ischemia is longer than 2 h, and mechanical damage of endothelium which can complicate reperfusion [13,22,23]. The complication rates may be reduced by using silicone coated sutures [13].

The intraluminal MCAO model is suitable for neuroprotection drug experiments because it produces a substantial amount of penumbra (salvageable tissue) in the first 60-90 minutes after onset [16]. Also, the location, volume, and temporal evolution of infarction are similar to those produced by proximal electrocoagulation of the MCA [16]. The suture is easily withdrawn, enabling investigators to study the aspects of reperfusion

The suture MCA occlusion model has recently been modified to induce ischemia in a magnetic resonance imaging (MRI) unit by remotely advancing the suture occluder [16]. This in-bore occlusion method has achieved a high reproducibility rate and enables investigators to monitor *in vivo *ischemic changes at pre-occlusion, acute, subacute and chronic post-occlusion time points [16]. Combined with multiparametric MRI techniques, the MCA occlusion model enables anatomic, diffusion, perfusion, and functional data to be obtained longitudinally and noninvasively in the same animal, making it a powerful tool for studying the pathophysiology of brain ischemia [23,24].

### Thromboembolic Model

Though thromboembolic ischemia can be induced by a photochemical approach, the most commonly used thromboembolic model is blood clot injection, first described in the dog by Hill et al. and later applied to the rat [25-27]. This model is of great interest to researchers because of its close resemblance to human ischemic stroke and its utility in evaluating thrombolytic therapies [28]. Thrombolytic therapy with recombinant tissue plasminogen activator (rt-PA) administered intravenously within 3 h of onset of ischemic stroke in select patients is the only FDA-approved treatment for human stroke and has been shown to improve neurological outcome [29]. Recently, there has been heightened interest in studying the efficacy of combining thrombolytic and neuroprotective agents in the treatment of stroke, giving thromboembolic animal models an increasingly important role in this respect [30].

Several disadvantages were common to the early thromboembolic models, such as diffuse and inhomogenous infarction in the MCA territory from microembolization to peripheral branches [27]. Additionally, spontaneous recanalization frequently occurred, making it difficult to study thrombolytic therapies [16]. Infarct sizes were variable, contralateral strokes were common, and ischemia caused by multiple small clots did not mimic typical clinical ischemic stroke [16]. Later, it was determined that size (length and diameter) and the biological characteristics of the blood clot (fibrin-rich) are crucial to the relevance and reproducibility of this model [16].

Busch *et al *developed a rat clot model that surmounted the above issues in which a single fibrin-rich autologous clot was injected to produce reliable occlusion of the proximal MCA, with consistent reduction of cerebral blood flow (CBF) and histological damage in the MCA territory seen [31]. No spontaneous thrombolysis was observed and, in separate experiments, thrombolytic therapy with rt-PA or prourokinase recanalized the occluded MCA [31,32]. Recently, Henninger *et al *used the embolic model in conjunction with multimodal MRI to investigate the pathophysiological mechanisms underlying the relatively rare clinical phenomenon of "spectacular shrinking deficit" in stroke patients [33].

In conclusion, the single fibrin-rich clot model induces reproducible infarcts in the MCA territory similar to those produced by the intraluminal MCA occlusion model. The clot model has the added advantages of bearing closer similarity to the mechanism underlying human ischemic stroke and better utility for studying thrombolytic therapy. Combined with modern imaging techniques, thromboembolic models have the potential to take the experimental study of stroke to new frontiers.

### Non-clot Embolus Models

Numerous compounds have been used to produce artificial emboli which are typically injected into the ICA, most commonly in rats [34]. Microsphere embolization is the most widely used model, with the severity of ischemic damage related to the number of emboli used [35]. The lesions require longer time to develop (24 hours on average) than in the intraluminal models, allowing for a larger therapeutic window for drug testing in microsphere models. However, the permanency of the ischemia does not simulate most clinical situations which limits the applicability of these models. Also, lesions are multifocal and have low reproducibility, though recent macrosphere models have resulted in more reproducible infarcts by increasing the diameter of the spheres and using less of them [36].

### Direct Surgical MCAO

Numerous techniques have been developed to surgically approach and occlude the MCA, with the rat being the species most widely used [13]. The orbital route is the least traumatic and, compared to procedures requiring craniotomy, results in less blood loss and artifacts [37]. Electrocautarization of the MCA results in permanent occlusion, whereas clipping or ligature snares enable reperfusion [38]. Occlusion of the MCA following a transient hypotension produces a larger infarct area [39]. Tandem occlusion of the distal MCA and ipsilateral CCA results in more reproducible infarcts [40]. Recently, a three-vessel occlusion model has been shown to produce reproducible and selective neocortical infarction [41]. Common to all of these variants, the procedure is always invasive and requires extreme surgical skill which limits their utility.

### Photochemically Induced Thrombosis

This model induces a cortical infarct by systemic injection of a photoactive dye in combination with irradiation by a light beam transmitted through the intact skull [42]. Oxidative damage to the endothelium caused by the altered dye leads to platelet aggregation in the irradiated area [43]. This model is used primarily in spontaneously hypertensive rats [44]. A disadvantage of this model is that vasogenic edema and blood-brain barrier breakdown occur within minutes which does not allow for the formation of penumbra--therefore, this model has been considered by many to be unsuitable for preclinical drug studies [44]. However, a new model overcomes this limitation and induces ischemia over a greatly extended time period, consistently producing a penumbra-like region [45]. This, combined with the ability to noninvasively and reproducibly induce infarct in any cortical location, are obvious advantages of the photochemical infarct model. A major disadvantage is the atypical features of the lesion (prominent vascular injury and early vasogenic edema) which are unlike human stroke [13].

### Endothelin-induced MCAO

Endothelin-1 (ET-1) is a natural peptide that causes vasoconstriction and several models use this as an agent to induce MCA stroke [13]. Invasive approaches have been largely replaced by stereotactic intracerebral injection of ET-1 adjacent to the MCA, which avoid complications of surgery [46]. When ET-1 is applied to the MCA there is a significant decrease of cerebral blood flow (CBF) in its territory, resulting in an ischemic lesion pattern similar to that of direct surgical MCAO [47]. This model may be useful in restorative drug studies. Notably, after a period (~20 minutes) of severe CBF reduction, there is a slow and progressive return of blood flow to normal with the rate being dose-dependent [47]. This can be disadvantageous source of variability unless the dose is carefully standardized in the experiment.

## Outcome Measures

There are several ways of measuring the severity of ischemic insult in animals: assessment of neurological status, pathological assessment, and in-vivo evaluation with magnetic resonance imaging (MRI). Most prior studies of MCAO focus on the acute phase of ischemia but since neuronal damage can occur days to weeks after insult, it is reasonable that new studies include evaluation of outcome measures both in the acute phase (1-3 days) and chronically (up to 4 weeks) [48].

### Assessment of Neurological Status

Many neurological deficits are difficult to assess in animals. Motor deficits are perhaps the easiest to quantify and simple measures of motor function are available in rodent models [49]. Refined tests that assess sensorimotor function include limb placing, beam walking, sticky label test, grid walking, and rotarod [50]. A number of cognitive tests examining learning and short term memory are available for rats, including the Morris water maze [51]. Combining neurological assessment with histological measurements is becoming more critical with the heightened interest in neuroprotective drugs, the effectiveness of which may be reflected more by subtle structural and chemical changes rather than changes in gross infarct volume [52]. This statement is supported by the fact that animal data suggest a poor correlation between reductions in infarct size with neurological or behavioral deficits [13]. Also of importance is the need to extend the period of testing of neurological status for at least 1 month post-insult in animal studies, per the recommendations of the STAIR Committee [14]. However, this may be difficult to implement as survival times are 48 hours or less in many animal stroke models [53].

### Pathological Assessment

In models of focal ischemia, the chief outcome has been the infarct volume, traditionally measured by quantitative histology. Among a number of histopathological methods, 2,3,5-triphenyltetrazolium chloride (TTC) and hematoxylin-eosin (H&E) staining are the two most commonly employed. TTC can be used to stain tissue much more rapidly, easily, and cheaply than H&E [54]. TTC is a colorless chemical that is reduced by mitochondrial enzymes into a compound that stains intact brain regions dark red whereas infarcted regions remain white. Studies show TTC staining to be reliable between 6 and 72 hours post-ischemia [13]. Prior to 6 hours, there may not be sufficient number of damaged mitochondria to create contrast between normal and infarcted tissue, and after 72 hours pathophysiologic inflammatory response often obscures the line of demarcation in the periphery of the damaged area [13].

Notwithstanding staining techniques, infarcts have complex shapes with sometimes indistinct margins, making it difficult to measure their volumes. Many methods have been developed to deal with these complexities, each with their own advantages and disadvantages. Perhaps one of the most important considerations to account for is the effect of vasogenic edema on infarct volume. Edema in the rat MCAO stroke model typically accounts for 20-30% of the total apparent infarct volume [55]. Separate measurement of "corrected" infarct volume (which accounts for edema) and "uncorrected" volume (which does not account for edema) is important because of the possibility that some interventions may reduce edema but not salvage brain tissue and vice-versa [13].

## Competing interests

The authors declare that they have no competing interests.

## Authors' contributions

Both KMS and MF performed background research and wrote the manuscript. All authors read and approved the final manuscript.
